# Clinical features and treatment of hypophosphatemia and associated complications induced by Phosphaturic mesenchymal tumors: A case series of six patients

**DOI:** 10.1016/j.bonr.2024.101822

**Published:** 2024-12-31

**Authors:** Guoqiang Lai, Wangsheng Zuo, Runmin Tang, Zengbo Lu, Dehai Shi

**Affiliations:** a Department of Joint and Trauma Surgery, The Third Affiliated Hospital, Sun Yat-sen University, No. 600 Tianhe Road, Guangzhou 510630, China; b Department of Pathology, The Third Affiliated Hospital, Sun Yat-sen University, No. 600 Tianhe Road, Guangzhou 510630, China; c Department of Spine Surgery, The Third Affiliated Hospital, Sun Yat-sen University, No. 600 Tianhe Road, Guangzhou 510630, China

**Keywords:** Phosphaturic mesenchymal tumor, Tumor-induced osteomalacia, Fibroblast growth factor 23, Hypophosphatemia, Renal impairment, Hyperparathyroidism

## Abstract

Phosphaturic mesenchymal tumor (PMT) is a rare benign mesenchymal tumor characterized by excessive secretion of fibroblast growth factor 23 (FGF23), leading to phosphate loss and systemic osteomalacia. Despite recent progress in PMT research, no consensus on diagnosis and treatment guidelines has been established. This case series describes the clinical and pathological features of six pathologically confirmed PMT patients treated at the Third Affiliated Hospital of Sun Yat-sen University from 2010 to 2024, aiming to provide new insights for the management of this condition. The patients, consisting of three males and three females with an average age of 44 years and follow-up periods of 0.5 to 4.5 years, presented primarily with muscle pain and lower limb weakness. One patient experienced loose teeth, and two had palpable, painless masses. One case developed hyperphosphatemia, tertiary hyperparathyroidism, and renal impairment after prolonged phosphate supplementation. Tumor localization was achieved using 18F-FDG or 68Ga-DOTATATE Positron Emission Tomography-Computed Tomography(PET/CT) and MRI, followed by complete surgical resection. Pathological examination confirmed PMT, and postoperative recovery was marked by significant symptom relief and normalization of serum phosphate levels. Two patients experienced recurrence within three years but showed no further recurrence following repeat surgery by the last follow-up. The diagnosis of PMT is challenging and may take years, potentially leading to complications due to inadequate treatment. Complete tumor resection remains the primary treatment, generally resulting in a favorable prognosis; however, long-term monitoring is essential to detect potential recurrences and initiate timely interventions.

## 1 Introduction

Phosphaturic mesenchymal tumor (PMT) is a rare metabolic bone disorder characterized by abnormally elevated levels of fibroblast growth factor 23 (FGF23), which results in impaired renal phosphate reabsorption and reduced synthesis of 1,25-dihydroxyvitamin D. This leads to hypophosphatemia and osteomalacia, a condition also known as tumor-induced osteomalacia (TIO) (Boland et al., 2018; Chong et al., 2011). Patients typically present with bone pain, fractures, and muscle weakness, significantly affecting their quality of life. The diagnosis of PMT is often delayed due to its nonspecific clinical manifestations and the frequently hidden location of tumor, causing patients to suffer for years before a definitive diagnosis is reached (Jan de Beur et al., 2023).

Early diagnosis and complete surgical removal of the FGF23-secreting tumor are crucial for reversing the disease. However, PMTs are often small, insidiously growing masses that are commonly located within bones or deep soft tissues, making them challenging to detect using conventional imaging modalities (Jiang et al., 2012). In recent years, functional imaging techniques such as 68Ga-DOTATATE PET/CT have demonstrated superior sensitivity and specificity compared to traditional imaging in localizing PMTs, providing critical guidance for accurate diagnosis and treatment planning (Bhalla et al., 2020; El-Maouche et al., 2016).

This study retrospectively analyzed six cases of confirmed PMT, investigating the impact of elevated FGF23 levels on calcium-phosphate metabolism and bone metabolism. Notably, we examined a case involving renal impairment and tertiary hyperparathyroidism induced by prolonged irregular phosphate supplementation. Through detailed analysis of these six cases, we aim to elucidate the complex pathophysiological mechanisms of PMT and their implications for clinical diagnosis and treatment, with the goal of enhancing awareness of clinicians of the disease, optimizing therapeutic strategies, and minimizing adverse outcomes associated with delayed diagnosis or inappropriate management.

## 2 Methods

This retrospective study analyzed the clinical data of six patients pathologically diagnosed with PMT at the Third Affiliated Hospital of Sun Yat-sen University between 2010 and 2024, all of whom underwent surgical treatment. General patient information, details of the diagnostic and treatment process, pre- and post-operative conditions, imaging findings, and pathological results were collected. Biochemical test data for all six patients were obtained from the hospital's laboratory system. Tumor localization was achieved in five patients using whole-body PET/CT, with subsequent confirmation through contrast-enhanced magnetic resonance imaging (MRI). In one patient, a palpable mass in the hand was clearly identified, and MRI with contrast enhancement indicated a PMT. Detailed surgical plans were formulated for each case, and complete tumor resection was performed, followed by pathological examination. All patients were followed up through outpatient visits or phone calls.

## 3 Results

This case series includes six patients, comprising three males and three females, with a mean age at presentation of 44 years (range: 30–66 years). Table 1 provides an overview of the patients' baseline characteristics and key biochemical parameters upon initial admission. The primary symptoms reported by the patients were diffuse musculoskeletal pain and bilateral lower limb weakness. Notably, Case 6 also presented with dental loosening, while Cases 4 and 5 had palpable, painless soft tissue masses. The average duration from symptom onset to diagnosis was 6.6 years, indicating a significant delay. None of the patients had a history of gastrointestinal disorders prior to admission.

**Table 1:** basic information and Laboratory results of patients on first admission to hospital.

	Reference range	Case1	Case2	Case3	Case4	Case5	Case6
Age/gender		66Y/F	30Y/M	63Y/F	38Y/F	52Y/M	38Y/M
Time*		4 years	1 year	5 years	3 years	4 years	11 years
Location		Mid-femur	Femoral head	Distal femur	Foot	Palm	Fibular head
Biochemical test							
ALP	35-135 U/L	564 ↑	232 ↑	162 ↑	216 ↑	290 ↑	296 ↑
IPHOS	0.74–1.52 mmol/L	0.29 ↓	0.56 ↓	0.35 ↓	0.54 ↓	0.54 ↓	0.48 ↓
Postoperative-IP	0.74–1.52 mmol/L	0.98	1.01	0.96	1.0	0.99	1.42
Ca^2+^	2.03–2.65 mmol/L	2.19	2.19	2.08	2.36	2.42	2.30
25(OH)D	》50 mol/L	59.8	49.7	57.5	51.5	84.2	NA
FGF23	23.3–95.4 pg/mL	NA	197.3 ↑	NA	NA	NA	NA
PTH	1.96–9.33 pmol/L	9.20	6.9	10.3 ↑	7.4	7.5	12.70 ↑
24 h U-P	16.1–42.0 mmol/24H	7.27 ↓	23.5	2 ↓	16.6	8.28 ↓	6.42 ↓
24 h U-Ca2+	2.5–7.5 mmol/24H	1.6	NA	NA	NA	2.55	1.45 ↓
eGFR	90-120 mL/min	99.16	139.28	107.68	129.46	114.76	NA
UA	90–420 umol/L	295	541 ↑	275	369	460 ↑	303.8
Recurrence		No	No	Yes	No	Yes	No

Initial laboratory tests revealed decreased serum phosphate, normal serum calcium, elevated alkaline phosphatase, and 25-hydroxyvitamin D levels that were either normal or mildly reduced, with normal liver and renal function. Mildly elevated parathyroid hormone levels were noted in Cases 3 and 6. The 24-h urinary phosphate excretion was not increased in any case. Regrettably, due to the limited technical resources available in our hospital, only Case 2 among the six patients underwent FGF23 testing before and after surgery. The samples were sent to an external laboratory for analysis. The FGF23 level was 197.3 pg/mL (23.3–95.4 pg/mL) 2.5 months prior to surgery, 171.1 pg/mL one week before surgery, and decreased to 28.2 pg/mL one week postoperatively.

**Fig. 1:**
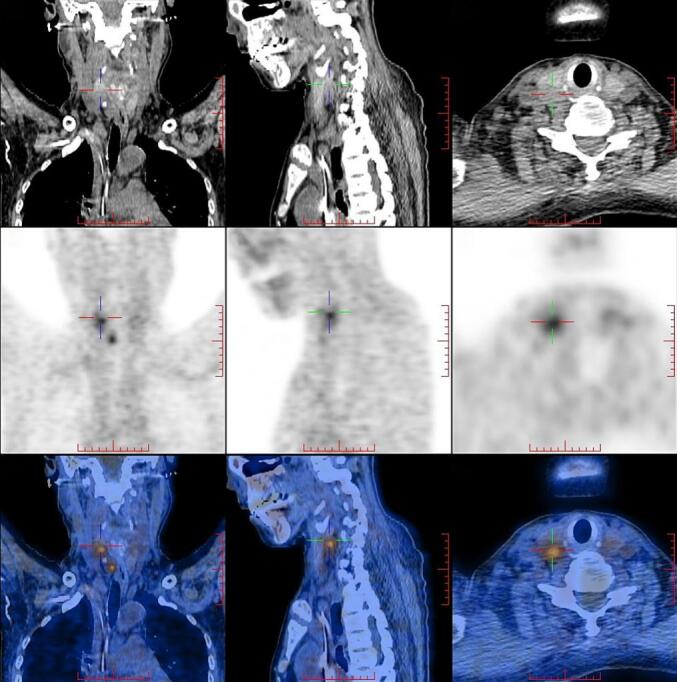
The PET/CT scan of Case 6 shows several slightly hypodense nodules in the parathyroid region, with the largest located on the right side, measuring approximately 16 × 11 mm. These nodules exhibit radiotracer uptake, with an SUVmax of approximately 4.3.

Preoperatively, among the six patients, two did not receive phosphate treatment. Case 3 took phosphate and calcitriol for two years, with parathyroid hormone levels slightly above the normal range but not meeting the diagnostic criteria for tertiary hyperparathyroidism, and renal function remained normal. Case 4 and Case 5 underwent short-term phosphate treatment for one week and 20 days, respectively, without any abnormalities in parathyroid or renal function. The condition of Case 6 was more complex. Case 6 warrants special attention, having been diagnosed with hypophosphatemic osteomalacia of unknown etiology in December 2011. At that time, routine blood tests, including liver and renal function, showed no abnormalities, and the patient underwent irregular sodium phosphate treatment for 12 years. Upon readmission in December 2023, the patient exhibited markedly elevated serum phosphate (3.66 mmol/L), creatinine (413.3 μmol/L), and parathyroid hormone levels (343.6 pmol/L), with an estimated glomerular filtration rate (eGFR) of 10.21 mL/min. The patient also had moderate anemia, with a hemoglobin level of 61 g/L. Complete blood count showed leukocytosis (white blood cell count 28.14 × 10^9^/L) and neutrophilia (absolute neutrophil count 26.9 × 10^9^/L, 95.6 %), with a C-reactive protein level of 385 mg/L, but no bacteria were detected in blood cultures. Metagenomic sequencing identified 73 reads of *Staphylococcus aureus* with a coverage of 45 %, and a chest CT scan showed bilateral pulmonary inflammation. Despite these findings, the patient did not exhibit typical symptoms of infection such as fever, cough, or urinary changes. Empirical treatment with meropenem led to normalization of blood counts and infection markers, although the eGFR remained reduced at 22.84 mL/min, and serum creatinine was still elevated (212.3 μmol/L), suggesting persistent renal impairment. Ultrasound revealed multiple hypoechoic lesions on the posterior aspect of the bilateral parathyroid glands, with the largest measuring 19 × 13 mm. Correspondingly, PET/CT showed several slightly hypodense nodules in the parathyroid region, the largest located on the right side, measuring approximately 16 × 11 mm, with radiotracer uptake and an SUVmax of 4.3, suggesting parathyroid hyperplasia (Fig. 1).Serum phosphate levels gradually decreased after discontinuing phosphate supplementation and reached 1.07 mmol/L prior to the surgical resection of a tumor located at the left fibular head. It was concluded that the patient's prolonged phosphate supplementation contributed to renal dysfunction and hyperparathyroidism, while impaired renal function led to phosphate retention and hyperphosphatemia.

**Fig. 2:**
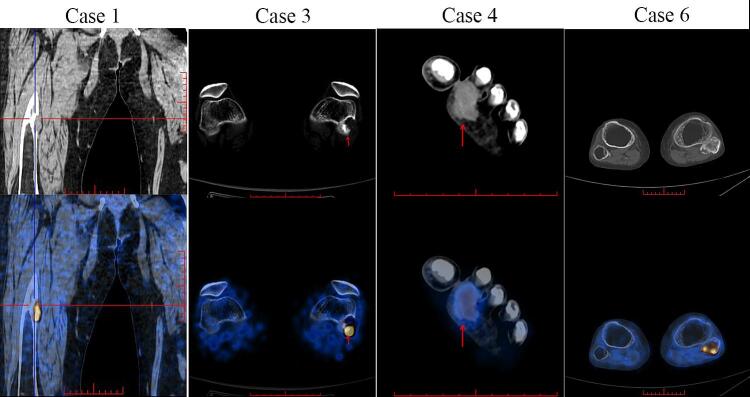
Positron Emission Tomography-Computed Tomography(PET/CT). In Case 1, 3, and 4, 68Ga-DOTATATE PET/CT revealed radiotracer-avid lesions located in the mid-right femur, lateral condyle of the left distal femur, and soft tissue of the left foot, with maximum standardized uptake values (SUVmax) of 17.8, 6.9, and 4.9, respectively. In Case 6, an 18F-FDG PET scan showed a radiotracer-avid lesion at the left fibular head, with an SUVmax of 4.7. For Case 2, imaging was performed at an outside hospital, and image data were not available. Case 5 did not undergo PET imaging.

Radiographs of all patients revealed varying degrees of osteoporosis. 68Ga-DOTATATE PET/CT in Cases 1, 3, and 4 detected radiotracer-avid lesions in the mid-right femur, distal left femur, and soft tissue of the left foot, with maximum standardized uptake values (SUVmax) of 17.8, 6.9, and 4.9, respectively (Fig. 2). In contrast, initial 18F-FDG PET/CT scans failed to detect tumors in Cases 2 and 6, although subsequent 68Ga-DOTATATE PET/CT performed two months later for Case 2 revealed a radiotracer-avid nodule in the left femoral head. For Case 6, an 18F-FDG PET/CT performed 11 years later identified a lesion in the left fibular head with an SUVmax of 4.7. Case 5, with a palpable painless mass in the right hand, underwent MRI with contrast enhancement, leading to a preliminary diagnosis of PMT without requiring whole-body PET scanning.

**Fig. 3:**
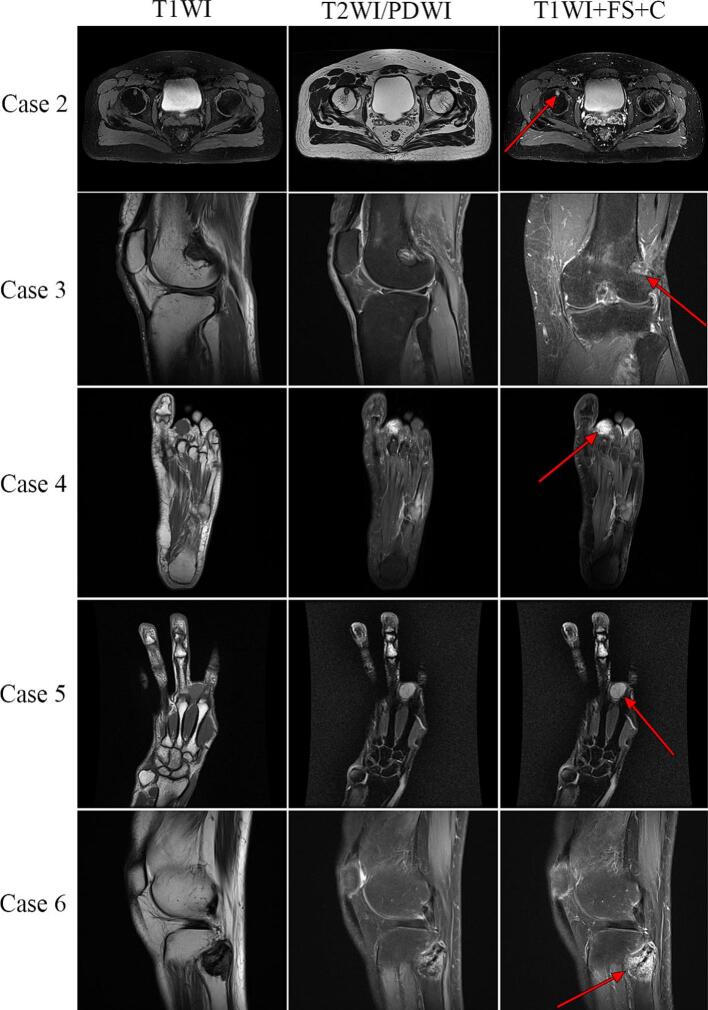
Magnetic Resonance Imaging (MRI). The tumors exhibited low signal intensity on T1-weighted imaging (T1WI), while on T2-weighted imaging (T2WI), they appeared with intermediate to high signal intensity (compared to the surrounding normal muscle tissue). On proton density-weighted imaging (PDWI), the tumors showed high signal intensity. Contrast-enhanced scans demonstrated either homogeneous enhancement (Case 2, 4, and 5) or heterogeneous enhancement (Case 3 and 6).The tumor sizes were 11 × 10 × 9 mm, 18 × 16 × 15 mm, 19 × 15 × 15 mm, 17 × 16 × 15 mm, and 30 × 30 × 25 mm, respectively.

MRI scans (plain and contrast-enhanced) were performed in five patients (Case 1 was excluded), revealing nodular or mass-like lesions in the tumor region. The tumor margins were well-defined in Cases 2, 4 and 5, but poorly defined in Cases 3, and 6. Tumors appeared hypointense on T1-weighted images (T1WI), hyperintense to intermediate on T2-weighted images (T2WI) relative to surrounding muscle, and hyperintense on proton density-weighted imaging (PDWI), with heterogeneous enhancement on contrast imaging (Fig. 3). The signal intensity was uniform across all sequences for Cases 2, 4, and 5, while Cases 3 and 6 exhibited heterogeneous signals, possibly due to necrosis, cystic degeneration, or hemorrhage in larger tumors.

Based on clinical symptoms, biochemical markers, and imaging findings, all patients were preliminarily diagnosed with PMT. Complete surgical resection of the tumors was achieved in all cases, and postoperative serum phosphate levels normalized in all six patients (Table 1). Symptoms of pain and weakness significantly improved following surgery.

Following the resection of the PMT, Case 6 continued to exhibit elevated parathyroid hormone levels despite normalization of serum phosphate levels. The patient remained unable to walk independently. Three months after discharge, the patient underwent a total parathyroidectomy at an outside hospital, with postoperative pathology confirming a diagnosis of parathyroid adenoma. Subsequently, the patient's mobility improved, and multiple follow-up serum phosphate measurements remained within the normal range.

Unfortunately, the patient's serum creatinine levels persisted between 210 and 270 μmol/L, indicating ongoing renal impairment. The patient is currently receiving regular outpatient nephrology care, including medication management, to prevent further deterioration of renal function.

**Fig. 4:**
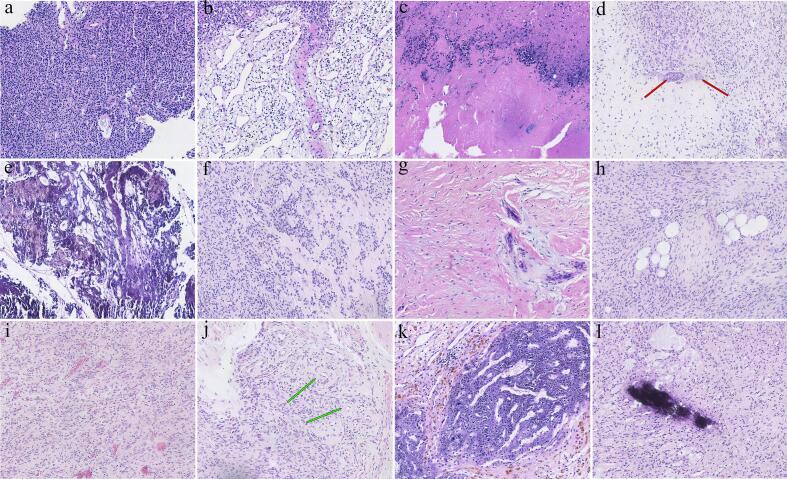
The pathological features of six cases of phosphaturic mesenchymal tumors. (a) Tumor cells diffusely arranged in sheets. (b) Microcystic structure. (c) Necrosis observed around tumor cells. (d) Scattered multinucleated giant cells (red arrows) surrounding the tumor cells. (e) Smoked background matrix appearance. (f) Myxoid degeneration of the stroma. (g) Fibrous tissue proliferation around tumor cells. (h) Mature adipose tissue interspersed with tumor cells. (i) Abundant thin-walled small blood vessels. (j) Abundant thick-walled blood vessels (green arrows). (k) Extensive hemosiderin deposition. (l) Focal calcification.

**Fig. 5:**
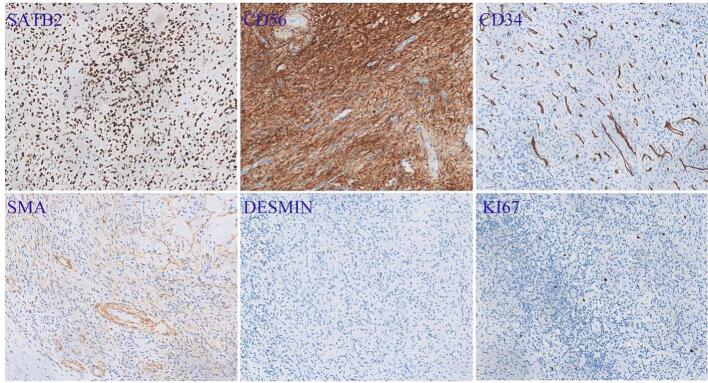
Immunohistochemical expression of phosphaturic mesenchymal tumors.

Histological examination of tumor samples from all six patients revealed spindle or plump spindle-shaped cells with mild nuclear atypia and rare mitotic figures (Fig. 4a), along with a characteristic “smoky” background matrix (Fig. 4e). Four cases showed microcystic structures (Fig. 4b) and multifocal calcifications (Fig. 4l), while three cases exhibited myxoid changes in the stroma (Fig. 4f), with prominent fibrotic tissue surrounding some tumor cells (Fig. 4g). Two cases had abundant small blood vessels, including thin-walled and thick-walled vessels (Fig. 4i, j), as well as interspersed mature adipocytes (Fig. 4h). One case showed small foci of necrosis (Fig. 4c) and scattered multinucleated giant cells infiltrating around the tumor (Fig. 4d). Immunohistochemically, all tumors expressed SATB2 and CD56, while CD34, SMA, and DESMIN were negative (Fig. 5). The proliferation index of tumor cells was low, at approximately 1 %. Based on clinical history and pathology, the final diagnosis in all cases was PMT.

Postoperative follow-up ranged from 6 months to 4.5 years, during which no tumor recurrence was observed in 4 patients. In Case 3, symptoms recurred 2 years after surgery, accompanied by a decrease in serum phosphate levels. After confirming the diagnosis, we performed a second tumor resection, and subsequent follow-up over the next 2 years showed no signs of recurrence. In Case 4, the patient underwent excision of a hand mass at another hospital in 2016, which was incidentally discovered. Although the patient had experienced generalized pain, the possibility of PMT was not considered at that time, and no pathological examination was conducted postoperatively, leaving the nature of the mass undetermined. In December 2019, the patient presented to our hospital with generalized pain. After a comprehensive diagnosis confirmed PMT in the same hand location, a complete tumor resection was performed. This case was thus regarded as a recurrence, and follow-up has continued for over 4 years without evidence of tumor relapse.

Red letters indicate abnormal results *Time from symptom onset to diagnosis ALP alkaline phosphatase PTH parathyroid hormone NA not available CREAT blood creatinine UA Uric Acid.

## 4 Discussion

Serum phosphate levels are regulated by intestinal absorption, glomerular filtration, renal tubular reabsorption, and the dynamic balance of phosphate between intracellular and extracellular spaces, as well as bone tissue. Disruption of these regulatory mechanisms over a prolonged period can lead to persistent hypophosphatemia, insufficient hydroxyapatite formation, and compensatory proliferation of osteoid structures (Kaneko et al., 2015; Ito et al., 2024). PMT cells excessively express FGF-23, which inhibits phosphate reabsorption in the renal tubules and the conversion of 25-hydroxyvitamin D to 1,25-dihydroxyvitamin D3, causing substantial phosphate loss in urine, leading to hypophosphatemia and osteomalacia (Bowe et al., 2001; Florenzano et al., 2021).

Clinically, PMT typically presents with bone pain, pathological fractures, muscle aches, and gait disturbances due to weakness. As the disease progresses, some patients may experience difficulty walking, multiple fractures, chest deformities, and respiratory difficulties (Moreira et al., 2006). Laboratory tests often reveal low serum phosphate levels, normal or reduced serum calcium levels, elevated alkaline phosphatase, and low 1,25(OH)2 vitamin D. The initial clinical symptoms and biochemical findings in our study of six cases were consistent with these observations.

FGF23 is the central pathogenic factor in tumor-induced osteomalacia (TIO), and elevated levels provide strong support for the diagnosis of phosphaturic mesenchymal tumors (PMTs). FGF23 testing is an essential diagnostic tool for determining the cause of hypophosphatemia, with high sensitivity and specificity. Armita Bahrami et al. used RT-PCR to detect FGF23 (Bahrami et al., 2009), finding positive results in 15 out of 16 PMT cases (94 %). Additionally, among 23 non-PMT samples, only 3 (13 %) tested positive for FGF23. These 3 positive samples included 2 chondromyxoid fibromas and 1 aneurysmal bone cyst, all of which were histologically and radiographically characteristic. This further underscores the necessity of integrating FGF23 testing results with all clinical, radiographic, and pathological findings before making a definitive diagnosis of PMT.

In our study, case 6 initially presented in 2011 without a definitive diagnosis, and after 12 years of irregular phosphate supplementation, the patient developed hyperphosphatemia, renal failure, and tertiary hyperparathyroidism. The possible mechanisms of renal damage are as follows:

1) Phosphate nephropathy: In a mouse study by Kazuhiro Shiizaki et al., FGF-23 was found to inhibit phosphate reabsorption in the renal tubules, increasing phosphate concentration in the tubular fluid. Once this concentration exceeds a threshold, microscopic particles containing calcium phosphate crystals appear in the tubular lumen, damaging tubular cells through interaction with TLR4 expressed on these cells. Persistent tubular damage induces interstitial fibrosis, reduces nephron numbers, and further elevates FGF-23 levels, perpetuating a vicious cycle that leads to progressive nephron loss (Broski et al., 2019; Letavernier and Drüeke, 2021). 2) Nephrocalcinosis: Oral phosphate can bind to calcium ions in the blood, forming insoluble calcium phosphate complexes, reducing free calcium levels. This hypocalcemic state stimulates parathyroid hormone secretion, which promotes calcium release from bones and increases calcium reabsorption in the kidneys while decreasing phosphate reabsorption, leading to increased urinary calcium excretion. Elevated concentrations of both calcium and phosphate in urine make it easier for calcium phosphate crystals to form in the renal tubules, resulting in nephrocalcinosis (Felsenfeld et al., 2015). This also explains the hyperparathyroidism seen in case 6. Markowitz reported a study in which five patients experienced varying degrees of renal dysfunction after oral phosphate supplementation. Renal biopsies revealed diffuse tubular injury and significant calcium phosphate deposition throughout the renal tubules, with lectin and immunohistochemical staining showing that calcium phosphate deposition was localized to the distal tubules and collecting ducts (Markowitz et al., 2004), strongly suggesting that oral phosphate was the cause of nephrocalcinosis.

Additionally, in case 6, whether *Staphylococcus epidermidis* infection could be a potential contributing factor to renal damage is worth considering. Laboratory findings and microbiological results suggest that the infection may have impacted renal function through a systemic inflammatory response. However, as the patient exhibited no infection-related symptoms, and urine tests showed no signs of urinary tract infection, we believe the infection might have played a secondary role in aggravating renal damage.

Case 6 also developed hyperparathyroidism, which may be explained by the following mechanisms based on previous research (Yang et al., 2023):

1) Prior to phosphate supplementation, FGF-23 inhibited the biosynthesis of 1,25(OH)2D, resulting in decreased serum calcium levels, which stimulated the secretion of parathyroid hormone.
2) After oral phosphate supplementation, the rapid rise in serum phosphate and the chelation effect of phosphate on calcium led to a reduction in serum calcium levels. The failure to promptly supplement calcium likely contributed to the development of hyperparathyroidism.
3) As the patient's renal function deteriorated, urinary phosphate excretion decreased, leading to elevated serum phosphate levels. Phosphate then bound to calcium in the blood, forming calcium phosphate, which caused hypocalcemia and further stimulated PTH secretion.

In contrast, Case 2, who underwent long-term phosphate supplementation combined with calcitriol treatment for two years, did not develop related complications. This finding aligns with the study by Ni et al. (2023), which included 202 patients, of whom 7 (3.5 %) eventually developed secondary hyperparathyroidism. In that study, the shortest duration from the onset of osteomalacia symptoms to PMT diagnosis was 7 years, and the minimum cumulative phosphate supplementation was 2190 g. The absence of tertiary hyperparathyroidism in Case 2 could be attributed to either an insufficient dosage of phosphate to trigger tertiary hyperparathyroidism or the concurrent use of calcitriol alongside phosphate supplementation.

After admission, phosphate supplementation was discontinued, and serum phosphate levels remained low until the tumor in the left fibular head was removed. Following the excision of the PMT and parathyroid glands, the patient regained the ability to walk, although renal function did not fully return to normal. This case underscores the need for close monitoring of calcium-phosphate metabolism and renal function in managing such patients, particularly during long-term phosphate supplementation, with the treatment regimen dynamically adjusted to balance efficacy and potential side effects.

Localization of PMT is highly challenging, with approximately 53 % occurring in bones, 45 % in soft tissues, and 3 % in the skin, making it difficult to detect through physical examination (Folpe et al., 2004). Functional imaging modalities such as 111In-pentetreotide, 99mTc-sestamibi, 18F-FDG PET/CT, and 68Ga-DOTATATE PET/CT can accurately identify the tumor's location (Broski et al., 2019; Hussein et al., 2021). PMT cells express various somatostatin receptors (SSTR1, 2A, 2B, 3, 4, 5), and 68Ga-DOTATATE, an SSTR antagonist, binds to these receptors, leading to receptor internalization and the accumulation of radioactivity within tumor cells (Jan de Beur, 2005). In our study, four patients underwent 68Ga-DOTATE PET/CT, all of which showed positive results, giving a detection rate of 100 %. Two patients who initially underwent 18F-FDG PET/CT failed to detect the tumor. Agrawal et al. compared the efficacy of 18F-FDG PET/CT and 68Ga-DOTATATE PET/CT in detecting primary tumors in suspected TIO cases, finding that 68Ga-DOTATATE PET/CT had superior sensitivity and specificity (Agrawal et al., 2015; Pelletier et al., 2017). While 18F-FDG PET/CT made significant contributions to the imaging evaluation of PMT before the widespread clinical application and approval of 68Ga-DOTATATE for TIO assessment (Broski et al., 2019), given the high costs associated with misdiagnosis and incorrect treatment, 68Ga-DOTATATE PET/CT should be the preferred imaging modality for clinically suspected cases of tumor-induced osteomalacia (Agrawal et al., 2015). Additionally, selective venous sampling to measure FGF23 concentrations, combined with MRI, may aid in tumor localization (Ito et al., 2024; Nasu et al., 2008; Takeuchi et al., 2004). Although these invasive methods have only been used in a few cases, their accuracy in localizing tumors still requires further validation through more studies.

The morphology of tumor cells in PMT is highly variable, often leading to misdiagnosis as other types of mesenchymal tumors. In all our cases, the tumors exhibited a characteristic “smudgy” basophilic matrix with areas of calcification or flocculent appearance. This matrix can stimulate the formation of multinucleated giant cells and proliferation of peritumoral fibrous tissue, necessitating differentiation from soft tissue giant cell tumors. In two cases, the HE-stained sections revealed abundant small blood vessels, which required differentiation from hemangiomas. Additionally, the presence of mature adipose tissue intermixed with spindle cells called for distinction from dermatofibrosarcoma protuberans. Immunohistochemical staining for CD34 was negative, helping to rule out both hemangiomas and dermatofibrosarcoma protuberans.

Morphologically, it was also important to differentiate PMT from metastatic carcinoma and osteosarcoma. Our immunohistochemistry results showed that the tumor cells diffusely expressed SATB2 and CD56. If the tumor occurs in the cranial region, meningioma should be considered in the differential diagnosis. In a few cases, the tumor exhibited high cellularity, significant atypia, and frequent mitoses (>5 per 10 high-power fields), suggesting an aggressive clinical behavior, often referred to as malignant phosphaturic mesenchymal tumor. Such cases may recur postoperatively, metastasize, and potentially result in patient mortality (Sidell et al., 2011; Morimoto et al., 2014).

The biological behavior of phosphaturic mesenchymal tumors is typically benign (Folpe et al., 2004), and malignant cases are extremely rare. Complete surgical resection is currently recognized as the most effective treatment strategy. After localization using whole-body PET/CT or MRI, a well-planned surgery can fully remove the tumor, which leads to rapid symptom relief and improved bone metabolism (Shane et al., 1997). In our series of six cases, all patients underwent surgical resection upon diagnosis, but two experienced recurrence. The reasons for recurrence may be related to incomplete resection of the tumor or undetected metastatic lesions. According to Taijun Hana's research, intraoperative measurement of FGF23 levels may help determine whether the tumor has been fully excised, given that FGF23 has a short half-life of approximately 18.5 min (Hana et al., 2017). However, we found that FGF23 testing has high technical requirements and lengthy turnaround times, limiting the feasibility of rapid intraoperative testing.

For tumors that are difficult to completely remove or have unclear margins, radiofrequency ablation can be an effective, minimally invasive option to destroy the tumor with low risk and side effects, offering an alternative to conventional surgery (Xian et al., 2021).

Literature suggests that octreotide, a somatostatin analog, can inhibit FGF23 secretion, thereby improving hypophosphatemia and bone abnormalities (Nair et al., 2017; Seufert et al., 2001). The high expression of somatostatin receptors on the surface of PMT cells makes peptide receptor radionuclide therapy a potential target for tumors that are inoperable or recurrent. Sandip Basu and Preeti Fargose successfully employed peptide receptor radionuclide therapy in treating a recurrent skull base phosphaturic mesenchymal tumor, achieving significant therapeutic effects (Basu and Fargose, 2016),roviding a valuable reference for cases where surgery is not an option.

Treatment with calcitriol and oral phosphate supplements can alleviate symptoms, but even when phosphate levels are maintained near normal, osteomalacia may persist (Nelson et al., 2003). These treatments require careful monitoring and dosage adjustment to avoid side effects. Burosumab, a fully human monoclonal antibody against FGF23, helps normalize phosphate metabolism and improve bone health by blocking excess FGF23. It has demonstrated good efficacy and tolerability within the limits of clinical trials and is the only FDA-approved medication for treating patients with TIO when the tumor is unresectable or cannot be localized, providing a crucial therapeutic option when surgery is not feasible (Jan de Beur et al., 2021).

In summary, through a retrospective review of six pathologically confirmed PMT cases and a literature review, we have summarized the diagnosis and treatment strategies for this rare condition. We also report one case of a PMT patient who developed hyperphosphatemia, renal impairment, and tertiary hyperparathyroidism due to long-term irregular phosphate supplementation—a scenario not previously documented in the literature. We hope this report will increase clinicians' awareness of this disease, enabling earlier and more accurate diagnoses to reduce the suffering caused by this destructive but curable condition. The limitations of our study include a small sample size of only six cases and a short follow-up duration; more case evidence and longer follow-up are needed to validate the surgical outcomes. After successful and complete tumor resection, the routine postoperative use of Burosumab may not be necessary. In cases of residual lesions or incomplete tumor resection, although large-scale studies specifically evaluating the combined use of Burosumab and surgery are lacking, case reports and small-sample studies have shown significant efficacy in patients with multiple surgical failures, suggesting its potential value in specific situations (Crotti et al., 2023; Crotti et al., 2021).

## 5 Conclusion

PMT is a rare solid tumor that can occur in various bones or soft tissues throughout the body. Its diagnosis is challenging and may take years, during which time improper treatment can lead to additional complications. The primary treatment approach is complete tumor resection, which generally results in a good prognosis. However, long-term monitoring of serum calcium and phosphate levels, along with regular follow-up, is essential to detect potential recurrences and initiate timely interventions.

## Glossary

PMT: Phosphaturic mesenchymal tumor
FGF23: fibroblast growth factor 23
TIO: tumor-induced osteomalacia
PET/CT: Positron Emission Tomography-Computed Tomography
MRI): magnetic resonance imaging
eGFR: estimated glomerular filtration rate
SUVmax): maximum standardized uptake values

## CRediT authorship contribution statement

**Guoqiang Lai:** Writing – review & editing, Writing – original draft, Visualization, Investigation, Formal analysis, Conceptualization. **Wangsheng Zuo:** Writing – original draft, Formal analysis, Conceptualization. **Runmin Tang:** Visualization, Investigation. **Zengbo Lu:** Investigation. **Dehai Shi:** Supervision.

## Informed consent statement

Written informed consent was obtained from the patients for the publication of this paper.

## Declaration of Generative AI and AI-assisted technologies in the writing process

During the preparation of this work the authors used [ChatGPT] in order to [grammar and language refinement]. After using this tool, the authors reviewed and edited the content as needed and take full responsibility for the content of the published article.

## Funding

This research did not receive any specific grant from funding agencies in the public, commercial, or not-for-profit sectors.

## Declaration of competing interest

The authors declare that they have no known competing financial interests or personal relationships that could have appeared to influence the work reported in this paper.

## Data Availability

Data will be made available on request.
